# Topographic and Bioclimatic Determinants of the Occurrence of Forest and Grassland in Tropical Montane Forest-Grassland Mosaics of the Western Ghats, India

**DOI:** 10.1371/journal.pone.0130566

**Published:** 2015-06-29

**Authors:** Arundhati Das, Harini Nagendra, Madhur Anand, Milind Bunyan

**Affiliations:** 1 Ashoka Trust for Research in Ecology and the Environment (ATREE), Bangalore, Karnataka, India; 2 Manipal University, Madhav Nagar, Manipal, Karnataka, India; 3 School of Development, Azim Premji University, Bangalore, Karnataka, India; 4 School of Environmental Sciences, University of Guelph, Guelph, Ontario, Canada; University of New England, AUSTRALIA

## Abstract

The objective of this analysis was to identify topographic and bioclimatic factors that predict occurrence of forest and grassland patches within tropical montane forest-grassland mosaics. We further investigated whether interactions between topography and bioclimate are important in determining vegetation pattern, and assessed the role of spatial scale in determining the relative importance of specific topographic features. Finally, we assessed the role of elevation in determining the relative importance of diverse explanatory factors. The study area consists of the central and southern regions of the Western Ghats of Southern India, a global biodiversity hotspot. Random forests were used to assess prediction accuracy and predictor importance. Conditional inference classification trees were used to interpret predictor effects and examine potential interactions between predictors. GLMs were used to confirm predictor importance and assess the strength of interaction terms. Overall, topographic and bioclimatic predictors classified vegetation pattern with approximately 70% accuracy. Prediction accuracy was higher for grassland than forest, and for mosaics at higher elevations. Elevation was the most important predictor, with mosaics above 2000m dominated largely by grassland. Relative topographic position measured at a local scale (within a 300m neighbourhood) was another important predictor of vegetation pattern. In high elevation mosaics, northness and concave land surface curvature were important predictors of forest occurrence. Important bioclimatic predictors were: dry quarter precipitation, annual temperature range and the interaction between the two. The results indicate complex interactions between topography and bioclimate and among topographic variables. Elevation and topography have a strong influence on vegetation pattern in these mosaics. There were marked regional differences in the roles of various topographic and bioclimatic predictors across the range of study mosaics, indicating that the same pattern of grass and forest seems to be generated by different sets of mechanisms across the region, depending on spatial scale and elevation.

## Introduction

Montane forest-grassland mosaics in the tropics form hotspots within hotspots of global biodiversity [1,2] supporting several endemic species [3] and are recognised centres of speciation for some taxa [4]. Both forest and grassland communities are characterised by high spatial turnover in species composition [2,5,6]. The complex and heterogeneous terrain on which they occur has the potential to provide important climatic microrefugia [7] for tropical biodiversity, especially under climate change.

As in other forest-grassland mosaics [8,9], there is evidence for climatic control on the distribution of forests and grasslands within mosaics in the Western Ghats. During the Pleistocene, forests spread over grasslands in warmer, wetter phases and contracted during cooler, dry periods [10,11]. During episodes of past climate change, topography mediated the extent of change between grass and forest, with forest expansion limited to sheltered valleys, possibly due to the effect of strong winds on steeper slopes and more exposed sites [11]. There appears to be a strong topographic effect on current vegetation pattern, with forest patches occurring in valleys, depressions and sheltered sites and grasslands occupying ridges, hill tops and exposed areas [12,13].

Topographic heterogeneity has complex effects on microclimate [7], impacting resource gradients for plants, such as sunlight, soil moisture and nutrients. At large spatial scales, elevation influences temperature and precipitation [14], while at finer scales, topographic position, terrain ruggedness and land surface curvature control the direction, rate and degree of convergence of flow of air and water, as well as mixing between the surface air and free-atmosphere layers [7]. Consequently, valleys are more weakly linked to regional temperature patterns and have greater diurnal temperature ranges than peaks or ridge tops [7]. Depressions and valley bottoms are also more frost-prone than elevated areas due to the pooling of cold air [7,15]. Aspect and slope influence solar insolation, thereby affecting local air temperature and soil-water balance through evapotranspiration [16,17]. Mid-lower slope positions usually have greater surface soil moisture levels than upland plateaus [16,18]. Topographically controlled hydrologic sorting of soil particles also affects soil texture, depth, pH and nutrient content at sites [19–21]. Cox et al. [20] and Lippok et al. [21] found that levels of pH and exchangeable calcium and magnesium increased from ridge to valley.

Elevation and topography affect the incidence, frequency and spread of disturbance processes such as fire [22–24]. Topographic orientation affects the flow of fire-bearing winds [22], while topographic heterogeneity increases frictional drag on winds and creates discontinuities in fuel-load and soil moisture [25]. Wood et al. [24] found that after accounting for vegetation type, topographic position, elevation and aspect were important predictors of fire occurrence. Forests occurring in mosaics with more flammable vegetation types are often located in topographic positions that inhibit the spread of fire such as near rocky outcrops, in valleys, depressions and on aspects sheltered from fire-bearing winds [22–24,26].

Although many studies have examined the effects of climate on forest-grassland mosaics, there is no quantitative study on the effect of topography on vegetation pattern across the full range of bioclimatic conditions in which these mosaics are found. A better understanding of the importance of various topographic features, how they interact with bioclimate and the spatial measurement scale at which they influence vegetation pattern could provide insights into mechanistic processes maintaining grasses and trees in tropical montane forest-grassland mosaics. Such an analysis could also help inform the management and conservation of these biologically important mosaics, especially in the face of climate change and other anthropogenic factors.

The main objectives of this analysis were to answer the following questions: i) to what extent can topography and bioclimate predict the pattern of occurrence of grass and forest patches within forest-grassland mosaics of the Western Ghats? ii) how does the relative importance of topography and bioclimate vary at different elevations? iii) what are the important interactions between topography and bioclimate for vegetation patterns? iv) what are the relevant measurement scales for topographic predictors that affect vegetation pattern within mosaics?

## Materials and Methods

### Study area

**Fig 1:**
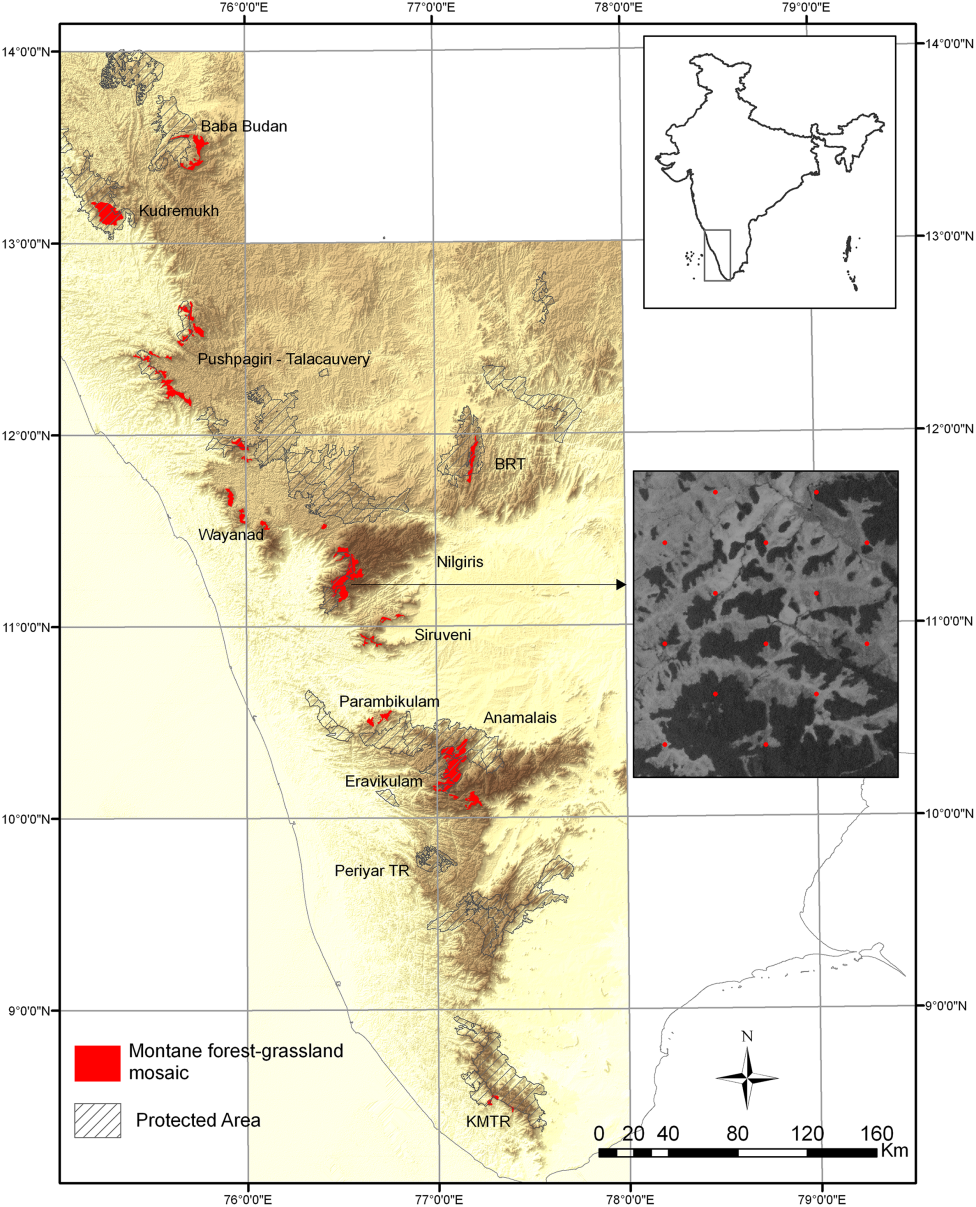
Map of study area. Map of the Western Ghats showing locations of montane forest-grassland mosaics and inset showing a section of an Indian Remote Sensing Satellite (P6) image of one of the study mosaics with sample points (superimposed in red) spaced 500m apart. Map created using ArcGIS (ESRI) software.

The study area encompasses montane forest-grassland mosaics of the Western Ghats between 8°22’-13°31’ latitude and 74°50’-77°30’ longitude (Fig 1). These habitats occur at the tops of the range; the largest expanses occur on high plateaus in the Nilgiris and Anamalai hills. Elevation for the study mosaics varies from approximately 500m to 2695m above mean sea level, while mean annual temperatures vary between 13–25°C, maximum warm season temperatures between 19–33°C, and minimum cold season temperatures between 0–20.5°C. Ground frost occurs at elevations above 2000m between November and February. Mean annual precipitation across the study mosaics ranges from approximately 800mm to above 6000mm. While most of the area receives rainfall primarily during the southwest monsoon (May–September), the eastern mosaics receive an increased proportion of rainfall during the northeast monsoon (October–December). Duration of the dry season ranges from one month in the southernmost mosaics to 4 months in the northernmost ones, and amount of rainfall decreases rapidly from west to east, especially at higher latitudes [3].

The mosaics consist of undulating grasslands interspersed with patches of stunted evergreen forests, locally known as ‘*shola*’. The boundary between forest and grassland is abrupt. *Sholas* have been classified as wet montane temperate forests [27], and as tropical montane cloud forests [28]. Trees are stunted (rarely above 15m tall), with relatively small, thick leaves, and trunks and branches covered with bryophytes and epiphytes [12]. Dominant families include Lauraceae, Rubiaceae, Myrtaceae and Symplocaceae [5,12]. The grasslands, which have also been called ‘shrub-savanna’, consist of grass, herb and shrub species [12]. These mosaics cover approximately 1% of the Western Ghats but are rich in endemic species, some of which are extremely rare. Cattle grazing and fire are common disturbances across these mosaics and there is very limited understanding of their impact on vegetation dynamics. These mosaics face a number of threats, including large scale land-use conversion, invasion by exotics and climate change [28,29].

### Delineation of forest-grassland mosaics and extraction of sample points

Mosaics were identified using high-resolution satellite imagery in Google Earth [30]. Polygons were digitised using visual interpretation and their locations and borders refined in consultation with three field biologists who have worked extensively in this habitat across the Western Ghats, in addition to the first author’s (AD) experience from previous fieldwork. This analysis represents the current extent of the mosaics and therefore underestimates the full range of topographic and bioclimatic conditions under which these mosaics naturally occur, for which there is insufficient data. In the Nilgiris and Anamalai hills, large areas of grassland, have been converted to exotic tree plantations [31]. The delineation of mosaic boundaries along lower slopes where *sholas* merge into continuous forest were necessarily subjective. These were drawn conservatively to restrict the study area to forests that occur within a matrix of grassland. Areas within and around Periyar Tiger Reserve were excluded because some of these grasslands appear qualitatively different from the grasslands in other mosaics (*pers*. *obs*.). All the mosaics depicted in Fig 1 were considered for this study.

A systematic sample of point locations covering the study area was created in ArcGIS v.10.0 [32], with a random start and spaced 500m apart (deemed adequate for spatial independence since habitat can change from grassland to forest several times over within this distance). The points were overlaid on high-resolution (~2.5m) imagery in Google Earth, and the habitat type of each point was classified as forest (“1”) or grassland (“0”) based on visual interpretation of the satellite image. Points falling within 30m of a forest-grassland edge (since the DEM has an average positional error of about 20m) were eliminated, as were points located in areas concealed by cloud cover. Points falling on rocky outcrops, water bodies and exotic tree plantations were also eliminated.

### Extraction and computation of topographic and bioclimatic predictors

A total of 1960 points of the original 2020 points were imported into ArcGIS 10.0 for analysis. A subset of points located in extreme topographic positions was used to confirm that spatial registration of imagery matched that of the DEM.

Selection of putative predictors of occurrence of forest and grassland within the mosaic was based on available published literature on the determinants of these patterns in such mosaics globally [10–12,15,22–24].

The ASTER Global Digital Elevation Model (GDEM) v.2 tiles (30m contour interval) [33] for the study area were used to extract the following topographic predictors in ArcGIS using Spatial Analyst and Topography toolbox: elevation, slope, transformed aspect, solar radiation [34], topographic position index [35], topographic convergence index [36], and surface curvature (Table 1).

**Table 1:** List of 27 topographic and bioclimatic predictors used for analysis and their ranges over the dataset.

Name	Code	Range	Description	Reference
Elevation	elev	455–2555m	Elevation of 30m pixel	[33]
Slope	slope	0.75–62.15 degrees	Local slope at 30m resolution	[33]
Ruggedness Index	rugged	4.24–157.46m	Terrain heterogeneity over a 3x3 cell neighborhood using a 90m DEM	[37]
Sine Aspect/ Cosine Aspect	sin.asp/ cos.asp	-1.00–1.00	E-W and N-S transformation of aspect at 30m resolution	[33]
Beers Aspect	Beers	0–2.00	SW-NE transformation of aspect at 30m resolution	[33]
Curvature 30m	curve30	-7.9–8.45	Combined across and along slope curvature, using a 30m pixel and 3x3 cell window	[33]
Curvature 90m	curve90	-3.3–4.04	Combined curvature, using a 90m pixel and 3x3 cell window	[33]
Local scale topographic position index	tpi3.10	-90.93–120.75m	Average difference in elevation between a focal cell and neighborhood defined using an annulus of inner radius 90m and outer radius 300m	[35]
Intermediate scale topographic position index	tpi10.34	-271.77–345.8 m	TPI using an annulus of inner radius 300m and outer radius 1020m	[35]
Landscape scale topographic position index	tpi10.67	-384.3–508.76 m	TPI using an annulus of inner radius 300m and outer radius 2010m	[35]
TCI 30m	tci30	0–366	Topographic convergence index using a 30m pixel	[36]
TCI 60m	tci60	-0.03–13.21	Topographic convergence index using a 60m pixel	[36]
TCI 90m	tci90	-0.56–11.02	Topographic convergence index using a 90m pixel	[36]
Distance to coast	coast.dist	25.27–174.11 km	Euclidean distance to coast line	
Solar radiation	solar	0.15–0.44 MJ/cm^2^/yr	Potential annual direct solar radiation based on latitude, slope and aspect.	[34]
Max. temperature warmest month	max.tmp	19–33°C		[38]
Min. temperature	min.tmp	4.1–20.5°C	Min. temperature in coldest month	[38]
Annual temperature range	anntmprng	10.5–18.2°C		[38]
Temperature seasonality	tmp.seas	891–1783	Temperature seasonality (standard deviation of temperature over the year*100)	[38]
Mean temperature dry quarter	meantmp.dry	11.9–25.5°C	Mean temp from Jan-Mar	[38]
Mean temperature warm quarter	meantmp.warm	13.9–27.5°C	Mean temp from Mar-May	[38]
Mean temperature cold quarter	meantmp.cold	11.2–24.3°C	Mean temp from Nov-Jan	[38]
Annual precipitation	annprec	754–6080 mm	Mean annual precipitation	[38]
Precipitation CV	prec.cv	50–140	Precipitation seasonality (coefficient of variation based on monthly precipitation values)	[38]
Warm quarter precipitation	warm.prec	165–893 mm	Avg. precipitation from Mar-May	[38]
Dry quarter precipitation	dry.prec	7–138 mm	Precipitation from Jan-Mar	[38]

TPI was calculated at local, intermediate and landscape scales (Table 1), defined by field-based observations of approximate distances between local hilltops and depressions and also wider valleys and peaks within some of the mosaics. TCI and surface curvature values for each sampling point were also extracted at different scales, by resampling the DEM to 60m and 90m resolutions. TCI was used as a proxy for soil moisture as it incorporates the upslope flow area above a given cell, identifying convergent points in the landscape which water would flow to. It is also a proxy for areas that are prone to cold air pooling and therefore frost [7,15]. Bioclimatic predictors (Table 1) were downloaded from the BIOCLIM global dataset, available at 1km resolution [38]. Since the study relied on remotely-sensed, publicly-accessible data sources, no field data collection was carried for this study. Therefore, neither were field study permits required nor did the research involve field studies of endangered or protected species. Ecologists who helped delineate mosaics and shortlist putative predictors based on extensive field experience are fully acknowledged.

### Classification trees and random forests

As the dataset comprised several highly correlated predictors likely to have complex interactions, a classification tree (CT) approach [39] was used to explore relationships between the response variable (forest or grass) and the predictors. CTs make no assumptions about underlying response functions and use recursive partitioning to split the data into increasingly homogenous subsets based on predictors. They are a powerful and intuitive method for visualising interactions between predictors [40,41].

CTs based on conditional inference [42] were constructed in R Statistical Software [43] package “party”. Conditional inference was chosen over the Gini index as a splitting criterion because the latter is biased towards predictors measured over larger scales or categorical predictors with many categories [44]. Conditional inference trees have a statistical stopping criterion, which prevents over fitting and eliminates the need for pruning [40].

A single CT built using all predictors and a random subset of the data was found to be quite unstable, with small changes in the training data yielding a different tree. Hence random forests [45] was used to assess: a) relative importance of the topographic and bioclimatic predictors and b) their combined ability to predict where forest and grass occur. In this approach a large number of CTs are built using random subsamples of both the data and the predictors. Each CT then “votes” for the final class of each data point and it is assigned to the class which has the majority of the vote. The portion of the data that was not used in building the CTs (i.e. “out-of-bag” data) is then used to assess the prediction error for the forest. Predictor importance is computed by randomly permuting values of each predictor in turn, thereby removing any association with the response, and then classifying “out-of-bag” samples using each CT and measuring the change in prediction accuracy after the permutation of that predictor [41,44]. This is then averaged across all CTs in the forest. Random forests compares favourably in terms of prediction accuracy against other approaches such as GLMs, GAMs and neural networks [41,46].

In order to maximize the size of the training dataset, a random subsample of two-thirds of the data was used in building the random forest and the remaining third “out-of-bag” data was used to assess its prediction error. The random forest algorithm was run 20 times with each run consisting of a 1000 conditional inference CTs [47]. For any given node within a tree, a subset of 5 randomly selected predictors was selected for splitting that node [42]. Overall prediction accuracy was assessed as the average of the ten random forest runs. Predictors of very low importance (near 0) were removed from the data. The list of predictors was then refined by beginning with the most important predictor and eliminating highly correlated (Pearson’s *r* > 0.7) predictors that were of lower importance. Random forests was then re-run with this smaller set of predictors and the cross-validated “out-of-bag” prediction accuracy reassessed as described above. Finally, to enhance our understanding of the effects and interactions between predictors, a conditional inference CT was built using the full dataset and the selected subset of predictors.

### Generalised linear modelling

Generalised linear modelling [48] was used as a different way of assessing predictor importance based on a) summed Akaike weights [49] and b) model averaged, standardised beta coefficients [50]. GLMs were also used to assess importance of interactions between predictors, many of which were identified using the conditional inference CTs.

To avoid the effects of collinearity and limit the number of predictors, and the number of models considered, we further eliminated predictors based on i) collinearity and ii) very low predictor importance (near 0) as demonstrated by the random forest analysis. Thus, the initial random forest analysis did feed into the GLMs, but not to the extent that it would seriously affect our final inference, since only obviously unimportant predictors were eliminated. Model-averaged estimates of each standardized beta coefficient were obtained as a weighted (using Akaike weights) average across all models containing that predictor. We also obtained unconditional standard errors (SE), which includes model selection uncertainty [49]. The 95% confidence intervals based on these SEs were examined to see if they straddled zero. Finally, Akaike weights were summed over all models containing a predictor as a measure of predictor importance. Because we required a balanced set of models where each predictor appeared in the same number of models [51] we fitted all combinations of the predictors, capped at a maximum of 6 predictors per model, allowing us to assess the importance of each predictor based on the summed Akaike weights [49]. This analysis was implemented in R using the package “MuMIn”.

We note that neither the CT nor GLM approaches as used by us represent confirmatory analyses [52] to test specific a priori hypotheses based on current understanding. While we did use available information to select putative predictors of the occurrence of forests or grasslands, the balanced set of models we assessed using GLMs is not a ‘candidate set’ (sensu [49]) where each model represents a specific scientific hypothesis, but a way to assess the importance of different predictors while ensuring that our inferences are not influenced by variable representation of different predictors within the set. Further we recognize that linear models do not represent causal relationships between the predictors and the response.

As the factors affecting vegetation pattern are likely to differ at low-medium versus high elevations (e.g. frost occurs only above a certain elevation), we assessed the change in the relative importance of topographic and bioclimatic predictors with elevation by repeating the above analyses for a subset of the data representing forest-grassland mosaics in the Nilgiris and Eravikulam plateaus above 1500m elevation (Fig 1). These high-elevation mosaics are also of particular conservation and management interest.

## Results

### Random Forests: Classification accuracy and predictor importance

The sample points (grass *n* = 1000, forest *n* = 960) cover a wide range of topographic and bioclimatic conditions (Table 1). The results of the random forest runs with the full dataset indicated a mean overall prediction accuracy for the “out-of-bag” data of 68.6%, (mean prediction accuracy for grass = 69.7%, forest = 67.6%). Random forest runs using only topographic predictors had lower mean prediction accuracy (66.5%; grass = 67.1%, forest = 65.8%).

**Fig 2:**
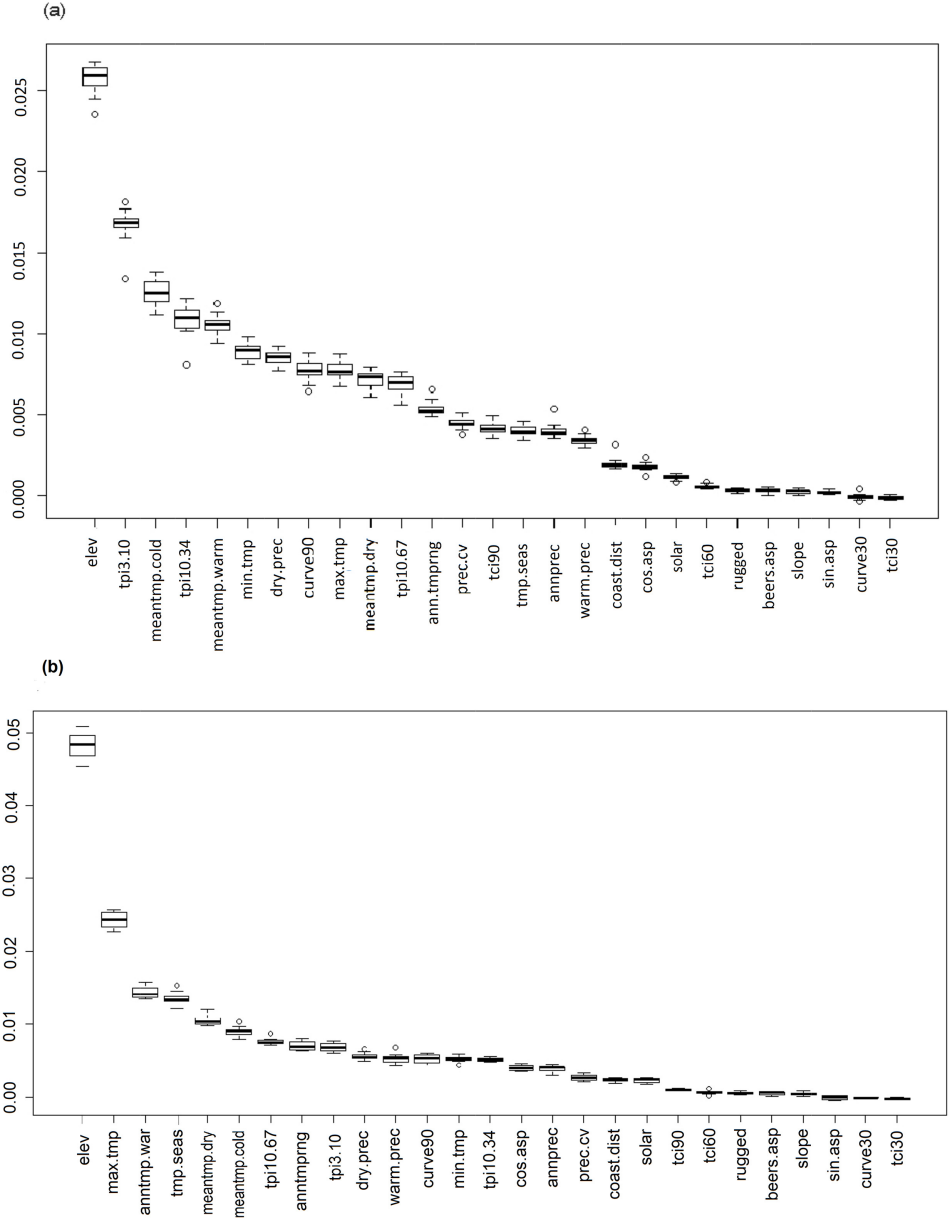
Boxplots showing distribution of permutation-based variable importance measures from random forests. Permutation-based variable importance measures for each predictor derived from multiple random forest runs for a) all mosaics b) Nilgiris and Eravikulam plateaus (> 1500m elevation). Please refer to Table 1 for explanation of predictor codes.

Permutation variable importance values averaged over 20 random forests runs (using the full dataset) indicated that elevation was the most important predictor, followed by local-scale TPI (tpi3.10). Predictors with the lowest importance values were TCI (30m pixel), curvature (30m pixel), sine aspect, slope and Beers aspect (Fig 2a). Therefore topographic features that influence convergence of air and water at the finest spatial scales in the landscape were not good predictors of vegetation pattern. Neither was slope exposure along an east-west axis nor a north-east to southwest axis.

Based on collinearity and the results of the permutation variable importance, the following subset of 11 predictors was chosen: elevation, local-scale TPI (tpi3.10), dry quarter precipitation, curvature (90m pixel), landscape-scale TPI (tpi10.67), annual temperature range, TCI (90m pixel), annual precipitation, distance to coast, cosine aspect and ruggedness. A marginal improvement in mean prediction accuracy for the full dataset was achieved over ten random forest runs for the “out-of-bag” data with this subset of predictors (69.2%; grass = 70.1%, forest = 68.3%).

For the Nilgiris-Eravikulam subset, (*n* = 783; 43% forest, 57% grass), the full set of predictors had an overall mean prediction accuracy of 72.1% (grass = 80.3%, forest = 63.9%), with many forest points being assigned to grass by the random forest algorithm. Elevation was the most important predictor, followed by maximum temperature of the warmest month (Fig 2b). Predictors related to temperature (most of which were very highly correlated with elevation (Pearson’s *r* ≥ 0.95)), gained importance over local-scale TPI. Landscape-scale TPI was more important than local-scale TPI at high elevations (Fig 2b). Cosine aspect, gained importance in the higher elevation dataset—and was negatively correlated with solar radiation (Pearson’s *r* = -0.67). Annual precipitation and dry quarter precipitation were less important at higher elevations (Fig 2b).

Based on collinearity and the results of the permutation variable importance measure, the following subset of 12 predictors was chosen for Nilgiris-Eravikulam mosaics: elevation, temperature seasonality, landscape-scale TPI (tpi10.67), annual temperature range, local-scale TPI (tpi3.10), dry quarter precipitation, curvature (90m pixel), cosine aspect, annual precipitation, distance to coast, TCI (90m pixel) and ruggedness. A higher overall mean prediction accuracy of 73.1% (grass = 81.4%, forest = 64.8%) was achieved with this subset of predictors and the “out-of-bag” data.

### Conditional inference classification trees: interpretation of predictor effects and possible interactions

**Fig 3:**
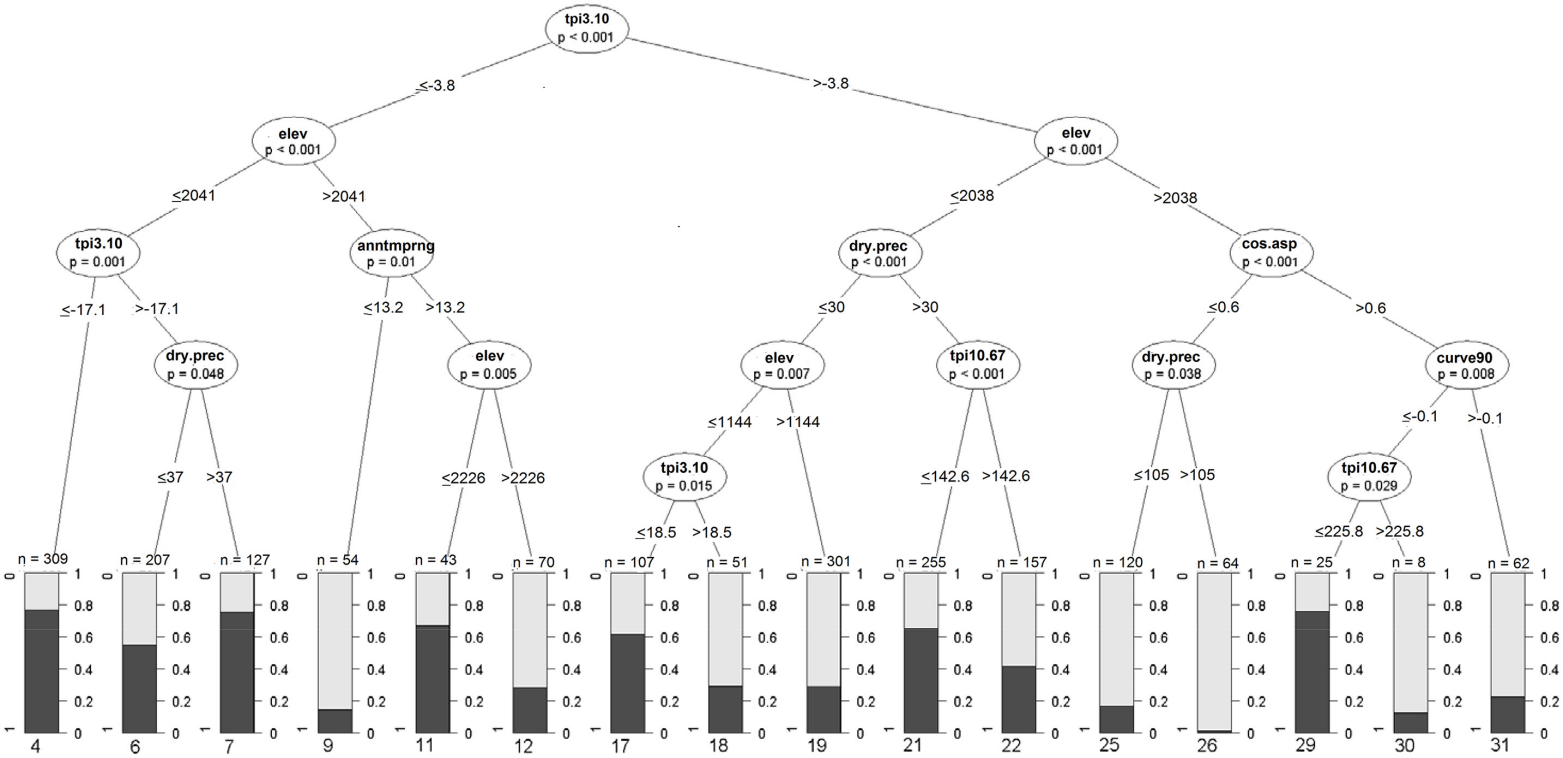
Conditional inference classification tree for forest and grassland points in forest-grassland mosaics of the Western Ghats. Node purity of terminal nodes depicted in bar charts with dark grey assigned to “forest” and light grey to “grass”. Terminal node identity numbers are given below each bar chart. For geographic breakdown of data points in each terminal node see S1 Fig. Please refer to Table 1 for explanation of predictor codes.

The conditional inference CT for the full dataset, using the subset of 11 predictors, indicated that local-scale TPI (tpi3.10) and elevation were important splitting variables followed by dry quarter precipitation and cosine aspect (Fig 3). The initial split made on tpi3.10, indicates that relatively small differences in local TPI are important. Another main split was based on elevation of about 2000m. The majority of points with higher tpi3.10 and elevations ≥ 2038m were classified as grass, with high node purity. Above 2000m, only 28% of the sample points were forest compared to about 50–60% in mosaics below 2000m. Slight, local topographic depressions (tpi3.10 ≤ -3.8m) below 2041m elevation were more likely to be identified as ‘forest’ compared to those above 2041m (Fig 3, Nodes 4–7 vs. Nodes 9–12), indicating an interaction between elevation and tpi3.10.

Sites between 1144–2038m elevation, with flat or elevated topographic positions and dry quarter precipitation ≤ 30mm,were predominantly grassland (Fig 3, node 19), as were most sites with high landscape-scale TPI (e.g. hilltops; tpi10.67 > 142.6m), in areas with dry quarter precipitation >30mm. Below 1144m, points with dry quarter precipitation < 30mm, that had large positive differences in local-scale TPI (tpi3.10 > 18.5m) (e.g. local ridge) were more likely to be grass. Therefore, where dry quarter precipitation was higher, landscape-scale TPI influenced whether a site held grassland or forest, whereas when dry quarter precipitation was low, differences in local-scale TPI had an impact on cover type (Fig 3).

**Fig 4:**
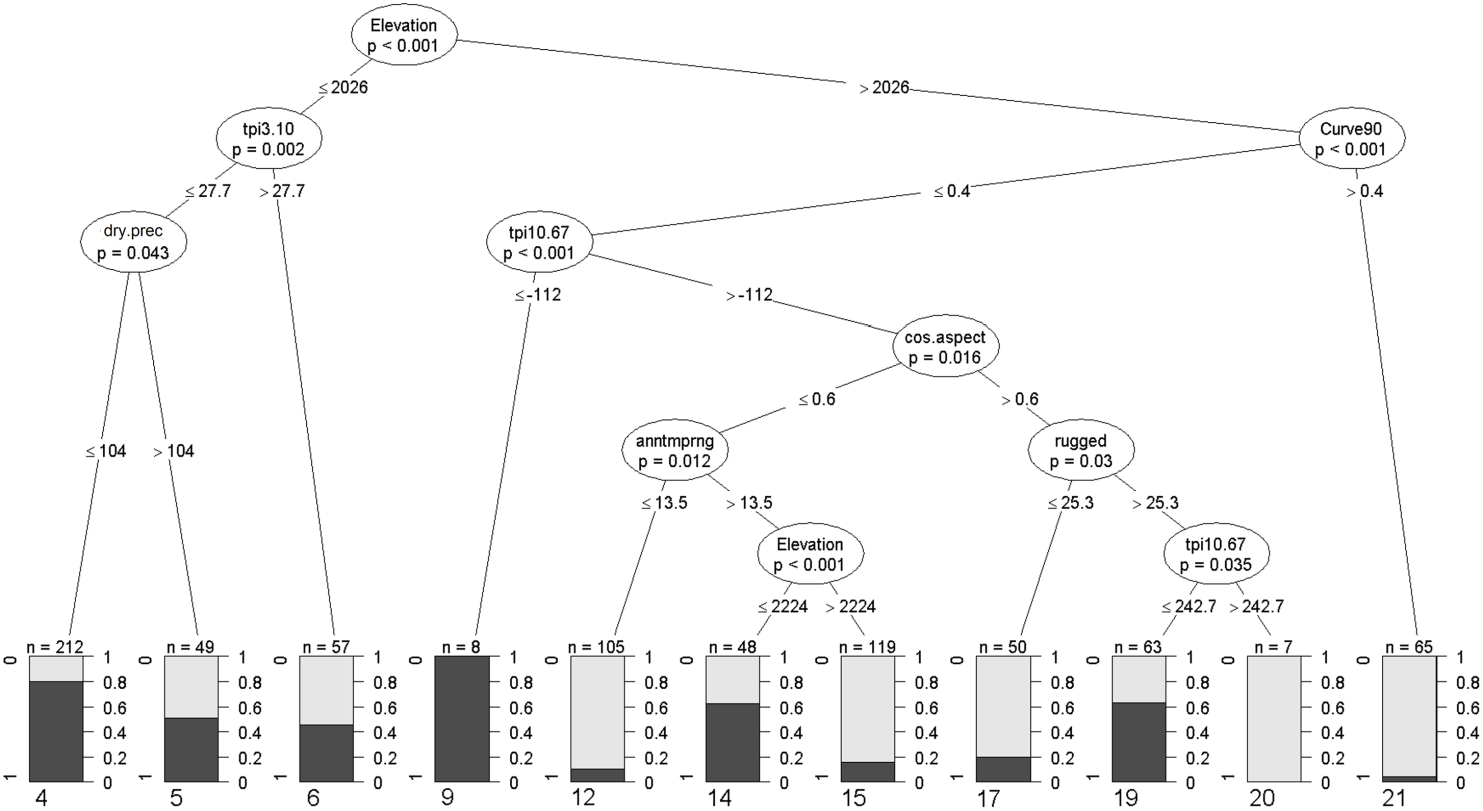
Conditional inference classification tree for forest and grass points in forest-grassland mosaics of the Nilgiris and Eravikulam (> 1500m elevation). Node purity of terminal nodes depicted as a bar chart with dark grey assigned to “forest” and light grey to “grass”. Terminal node identity numbers are given below each bar chart. Please refer to Table 1 for explanation of predictor codes.

There seemed to be complex interactions among topographic variables as well as between elevation and annual temperature range (Figs 3 and 4). For higher elevation points, elevation, annual temperature range, cosine aspect, curvature (90m pixel) and landscape-scale TPI (tpi10.67) were important splitting variables.

The CT built from the subset of 12 predictors for Nilgiris-Eravikulam, showed that between 1500–2555m, elevation was the most important splitting variable, followed by local-scale TPI at lower elevations and local curvature (curve90) at higher elevations (Fig 4). Points between 1500m–2026m were mostly classified as forest, particularly when they had lower tpi3.10 values. Grasslands in this elevation band were not classified well, as shown by the high level of node impurity (Nodes 5 and 6 in Fig 4). Points > 2026m elevation and with convex curvature (i.e. curve90 > 0.4) were classified as grass with high node purity (Node 21 in Fig 4). Points > 2026m elevation were classified as forest with high node purity only when they lay on flat or concave local curvature and at relatively low landscape-scale TPI, i.e. deeper valleys (Node 9 in Fig 4). Above 2026m, forest points fell mostly on NE to NW aspects. More south-facing sites with annual temperature range >13.2°C, above 2224m elevation, were classified as grass with very high node purity (Node 15 in Fig 4).

### Generalised linear models: predictor importance

The analysis on the full dataset (*n* = 1960) using 11 predictors and interactions between some of the predictors, indicated that the two best models had the following predictors for occurrence of forest: elevation, dry quarter precipitation, local-scale TPI, annual temperature range, interaction between annual temperature range and dry quarter precipitation, curvature (90m pixel) and cosine aspect. Predictors with highest summed Akaike weights were: elevation, dry quarter precipitation, local-scale TPI, annual temperature range, an interaction between annual temperature range and dry quarter precipitation, curvature (90m pixel) and cosine aspect (Table 2). Beta coefficients of the top three predictors supported their importance.

**Table 2:** Assessment of the importance of predictors used to model forest points within montane forest grassland mosaics across the Western Ghats.

Predictor^a^	Summed Akaike weight	Standardized, model-averaged beta (SE)	Model averaged 95% CI
elevation	~1	-1.014 (0.094)	-1.199: -0.829
dry.prec	~1	0.92 (0.117)	0.69: 1.15
tpi3.10	0.999	-0.449 (0.085)	-0.616: -0.283
anntmprng	0.999	0.36 (0.08)	0.204: 0.516
anntmprng:dry.prec	0.999	0.323 (0.056)	0.214: 0.432
curve90	0.579	-0.259 (0.063)	-0.383: -0.136
cos.aspect	0.379	0.204 (0.05)	0.107: 0.301
elevation:tpi3.10	0.029	0.19 (0.055)	0.082: 0.298
annprec	0.008	-0.304 (0.099)	-0.498: -0.109
coast.dist	0.002	0.202 (0.087)	0.031: 0.372
elevation:dry.prec	0.0009	-0.173 (0.077)	-0.323: -0.022
tci90	0.0003	0.091 (0.056)	-0.018: 0.2
tpi10.67	0.0002	-0.091 (0.062)	-0.211: 0.03
dry.prec:tpi3.10	0.0001	0.061 (0.056)	-0.05: 0.171
rugged	0.0001	0.003 (0.05)	-0.096: 0.102
dist.coast:elevation	0.0001	-0.571 (0.113)	-0.792: -0.35
cos.aspect:elevation	~0	0.163 (0.051)	0.063: 0.263
elevation:tci90	~0	-0.209 (0.055)	-0.318: -0.1
curve90:elevation	~0	0.051 (0.058)	-0.063: 0.165
dry.prec:tci90	~0	-0.108 (0.06)	-0.226: 0.01
cos.aspect:coast.dist	~0	0.062 (0.052)	-0.04: 0.164

Columns show summed Akaike weights, standardized beta coefficients averaged across models and unconditional standard errors (SE) in parentheses and 95% confidence intervals (CI) based on the unconditional SEs.

^a^Please refer to Table 1 for explanation of predictor codes

Important bioclimatic predictors not highly correlated with elevation were dry quarter precipitation and annual temperature range (Table 2). Both show a strong latitudinal gradient, with dry season precipitation decreasing and annual temperature range increasing with latitude (Pearson’s *r* = -0.93 and 0.72, respectively).

The analysis for the Nilgiris-Eravikulam mosaics (*n* = 783) indicated that the best model based on AIC had the following predictors: elevation, annual temperature range, curvature (90m pixel), cosine aspect, distance to coast, and an interaction between cosine aspect and distance to coast. These predictors also had the highest summed Akaike weights (Table 3). Beta coefficients of the top five predictors supported their importance. Compared to the full data set, the importance of annual temperature range, cosine aspect, curvature (90m pixel) and distance to coast increased, while that of local-scale TPI, dry quarter precipitation and its interaction with annual temperature range decreased.

**Table 3:** Assessment of the importance of predictors used to model forest points within montane forest grassland mosaics above 1500m elevation in the Nilgiris and Eravikulam.

Predictor^a^	Summed Akaike weight	Standardized, model-averaged beta (SE)	Model averaged 95% CI
elevation	1	-1.529 (0.14)	-1.804: -1.254
anntmprng	0.999	0.819 (0.169)	0.489: 1.15
cos.aspect	0.999	0.42 (0.088)	0.247: 0.592
curve90	0.996	-0.489 (0.117)	-0.718: -0.259
coast.dist	0.537	0.248 (0.112)	0.029: 0.467
cos.aspect:coast.dist	0.377	0.269 (0.091)	0.09: 0.447
tci90	0.223	-0.189 (0.106)	-0.397: 0.019
tpi3.10	0.187	-0.214 (0.111)	-0.433: 0.004
elevation:curve90	0.172	-0.191 (0.094)	-0.375: -0.006
elevation:tci90	0.094	-0.319 (0.121)	-0.557: -0.082
dry.prec	0.058	0.308 (0.233)	-0.149: 0.765
anntmprng:curve90	0.057	-0.152 (0.111)	-0.37: 0.066
tpi10.67	0.053	-0.14 (0.106)	-0.349: 0.068
elevation:tpi3.10	0.039	0.227 (0.102)	0.026: 0.428
cos.aspect:elevation	0.035	0.098 (0.1)	-0.098: 0.294
annprec	0.025	-0.043 (0.11)	-0.259: 0.173
anntmprng:elevation	0.024	-0.054 (0.136)	-0.321: 0.213
rugged	0.023	0.029 (0.093)	-0.154: 0.213
coast.dist:elevation	0.014	0.182 (0.122)	-0.057: 0.422
cos.aspect:tci90	0.010	-0.167 (0.087)	-0.338: 0.003
cos.aspect:tpi3.10	0.009	0.13 (0.095)	-0.056: 0.316
dry.pre:elevation	0.001	0.016 (0.134)	-0.248: 0.28
anntmprng:dry.pre	0.001	0.025 (0.201)	-0.371: 0.42

Columns show summed Akaike weights, standardized beta coefficients averaged across models (unconditional standard errors SE in parentheses) and 95% confidence intervals (CI) based on the unconditional SEs.

^a^Please refer to Table 1 for explanation of predictor codes

### Interactions between topographic and bioclimatic predictors

The conditional inference CTs indicated complex interactions between elevation, local-scale TPI, dry quarter precipitation and annual temperature range (Fig 3); however the results of the GLM provided only limited support for this (Table 2), possibly due to the limited ability of GLMs to model the complex interactions indicated by the CTs. Other than the interaction between annual temperature range and dry quarter precipitation, none of the interaction terms tested on the full dataset was rated as important predictors by the GLMs. However, the 95% confidence intervals for standardised beta coefficients of the interactions between elevation and local-scale TPI, elevation and dry quarter precipitation and elevation and distance to coast did not straddle zero, indicating some support in the models (Table 2).

For Nilgiris-Eravikulam mosaics, the interaction between distance to coast and cosine aspect was the most important of the interactions (Table 3), however this was not detected by the CT (Fig 4). There was some support for interactions between elevation and curvature (90m pixel) and elevation and TCI (90m pixel). The interaction between elevation and curvature (90m pixel) had a higher summed Akaike weight but a smaller beta co-efficient than the interaction between elevation and TCI (90m pixel) (Table 3).

### Scale of topographic control on vegetation pattern

The results indicated that topographic pattern within a 300m neighbourhood, was important for vegetation pattern, as measurement of topographic features at this scale (i.e. tpi3.10, curve90 and tci90) was the most relevant for prediction of forest and grassland patches (Fig 2, Table 2). Local hills and depressions (tpi3.10) and surface curvature (curve90) seemed to be more influential than prominent peaks and valleys (tpi10.67), though importance of the latter increased in mosaics above 2000m (Fig 2b, Table 3).

## Discussion

Topography and bioclimate were able to predict the occurrence of grass and forest within forest-grasslands mosaics of the Western Ghats with approximately 70% accuracy. Prediction accuracies were higher for grass compared to forest and for mosaics at higher elevations (1500–2000m). Topography alone was able to predict forest and grassland pattern well, however inclusion of bioclimatic predictors that captured latitudinal gradients in climate improved predictive accuracy.

The misclassification of high elevation forest points may be due to the fact that the predictors measured at the given scales were unable to correctly classify many of the smaller sized (< 2 ha) forest patches that occur in these mosaics. The inclusion of microclimatic data [7] could improve classification accuracy for forest patches at higher elevations. Classification accuracy was lower for the lower elevation mosaics between 12° to 13°12’ latitude that lie closer to the coast (S1 Fig), indicating that the predictors chosen for this study were unable to characterize vegetation pattern in these mosaics as well.

Overall, the instability found in the CT analysis indicates regional differences in predictor effects and interactions between topography and bioclimate across the range of these mosaics. Hence the same pattern of grassland and forest seems to be generated by different sets of mechanisms across the Western Ghats.

### Elevation and bioclimate

Sankaran et al. [53] demonstrated that mean annual precipitation is the main driver of tree cover in African savannas with low rainfall. In tropical montane forest-grassland mosaics with high mean annual precipitation, elevation is an important predictor of vegetation pattern. Specifically, in the Western Ghats, there seems to be a shift in pattern at about 2000m elevation, above which there is a much lower occurrence of forest. This indicates a climatic effect on tropical evergreen tree growth and survival [54]. Since bioclimatic predictors related to temperature showed the strongest correlation with elevation, it seems that temperature, rather than precipitation, is the main proximate climatic driver of pattern in high elevation mosaics. The relative importance of elevation and correlated temperature variables increases with elevation, further supporting the view that temperature has an important influence on vegetation pattern in Nilgiris and Eravikulam [11].

Temperature is the major limiting factor for tree growth at treelines [55]. While the mosaics of the Western Ghats are well below the climatically defined treeline in the tropics [54,55], lower average air and soil temperatures above 2000m may limit most tropical tree species’ establishment and survival [54,56]. This is supported by the observation that tree species composition within *shola* patches in the Western Nilgiris shows high turnover between 1900-2000m (A. Das unpublished data), with an increasing component of upper montane taxa and frost-resistant species above 2000m [57]. Ohsawa [54] posits that the thermal limit for lower montane tropical trees occurs at 2,500m in equatorial mountains, with mean annual temperatures of 12°C and 10°C in the coldest month. Mosaics above 2000m have mean annual temperatures of 14.1°C and an average minimum temperature of 7.1°C in the coldest month. Caner et al. [11] report that temperatures during the Last Glacial Maximum were about 5°C lower than present day in the Nilgiris, with grassland probably covering most parts of the plateau above 1800m.

The climate signal implied by this altitudinal shift in the occurrence of forest lends support to the role of frost in restricting forest above 2000m [13]. It is unlikely that the predominance of grassland, noted in historical accounts and palaeoclimatic reconstructions [10,11,13], can be explained solely by increase in the frequency of disturbance [58] in these mosaics, when compared to those at lower elevations. There is evidence for a strong role of disturbance processes in shaping vegetation pattern in lower elevation forest-grassland mosaics from other parts of the world [59]. However, fire frequency in the high-elevation mosaics should be lower, as they are sparsely populated and have been managed as protected areas for the last 30–40 years [60].

Mohandass and Davidar [57] found evidence to suggest that *sholas* expand into grassland through succession beginning with establishment of frost-resistant woody species in grasslands, and subsequently creating suitable conditions for establishment of lower montane species. An analogous process of forest expansion occurs in subtropical forest-grassland mosaics of Southeastern Brazil, where fire has a major influence on vegetation pattern [61].

In mosaics below 2000m, dry season precipitation is an important predictor of forest occurrence. There is some evidence that its influence is mediated by both topographic position and elevation (Fig 3). This could imply a fire-related mechanism in maintaining grasslands at middle and lower elevations, where lower dry season precipitation allows for greater incidence and spread of fire [2]. This could prevent forests from establishing in topographic positions that they might otherwise occupy in mosaics with higher dry season rainfall.

Finally, the importance of the interaction between dry quarter precipitation and annual temperature range on the presence of forest implies that forests at mid-elevation (approx. 1000–2000m) areas of the central Western Ghats such as the Nilgiris plateau and the Siruveni hills could be more strongly influenced by these bioclimatic factors than elsewhere (Fig 1).

### Topographic effects

As expected, topographic position is an important predictor of vegetation pattern in these mosaics—certainly as a main effect, possibly also in interaction with elevation. Sites with lower topographic position were associated with forest. This could indicate an effect of soil moisture, especially in mid-lower elevation mosaics. Local depressions and valleys are likely to be wetter and therefore less vulnerable to fire [24]. However, local topographic depressions above 2000m were more likely to contain grass than forest, possibly due to waterlogging and frost [7,15,62]. At elevations above 2000m, relative topographic position at the landscape scale (300-2010m neighbourhood) and concave surface curvature are more important predictors of forest than local topographic position, corroborating field observations. Larger patches of forest in these mosaics are often confined to sheltered valleys (*pers*. *obs*.). The greater importance of local curvature in high elevation mosaics could reflect the influence of concave depressions along hill slopes [13] that provide adequate soil moisture while allowing for drainage, thus preventing the negative effects of waterlogging or frost on tree growth [15,62].

It is interesting that local topographic position and surface curvature were more important predictors of vegetation pattern than topographic convergence, as previous studies have found topographic convergence to be an important predictor of forest occurrence [15,63].

Aspect, specifically northness, is an important predictor of forest in high elevation mosaics, which has been attributed to differences in radiation exposure [15]. Bunyan et al. [63] find a similar result for these mosaics. The orientation of topography in Nilgiris and Eravikulam could result in southern slopes receiving higher solar radiation, possibly implying that water stress or desiccation is limiting for trees [7]. Drier southern aspects could also be more prone to fire. Wood et al. [24] demonstrated that rainforests occurring in a matrix of moorland in Tasmania were restricted to southern aspects and topographic positions very similar to those found in this study, as these places formed fire refugia. Using a modelling approach, Blanco et al. [59] also found that southern aspects were more conducive to spread of forest patches in a matrix of fire-prone, lower elevation grasslands in Southern Brazil. Prevailing wind direction may be important as southern and western slopes are affected by strong monsoon winds, while northern and eastern aspects are more sheltered [11]. The importance of northness and distance to coast in predicting forest at high elevations provides some support for this (Table 3). The role of aspect in these mosaics should be investigated further using field experiments and measurement of microclimatic conditions.

Fletcher et al. [62] show that a transition from one vegetation state to another can be generated by one set of factors and regulated by another. The initial climatic constraint on forests in high-elevation mosaics could be maintained under present climate by cumulative effects of low temperature, frost, fires and wind. Topographic heterogeneity can modulate the intensity and spread of each, possibly helping to create sharp boundaries [22–24]. Vegetation pattern in these mosaics could be maintained by a group of interacting factors acting in a spatially heterogeneous manner- determined by topography- and in feedback with vegetation type [23], rather than by a single limiting mechanism—be it frost [13], fire, grazing [58], wind [11] or soil [64]. This analysis provides strong support for topographic control on processes maintaining vegetation pattern in these systems.

### The need for data

A major limitation of this study is that it does not consider resource and disturbance gradients such as soil characteristics, fire and grazing, that have been found to be critical in shaping vegetation pattern in other forest-grassland systems [8,24,61]. Much of the misclassification of vegetation pattern, particularly at lower elevations, could be due to the effects of these factors. Lower elevation mosaics are more exposed to anthropogenic disturbance. At present the required data are not available at relevant scales across the study area.

There is an urgent need for accurate, high resolution spatio-temporal datasets on disturbance, particularly fire, across the study area. We found that global satellite-derived burned area products largely underestimated fire frequency in several mosaics, precluding their use in this analysis.

### Implications for management and conservation

The results indicate that ongoing *shola* restoration efforts by State Forest Departments in mosaics above 2000m, should be focused on north-western to north-eastern aspects. Given past conversion of large areas of grassland into exotic tree plantations, recent global trends of forest expansion into grasslands [2,9] and palaeoclimatic trends of forest expansion during warming climates [10], it is important that restoration of *shola* does not take place at the expense of existing grasslands [65].

As mosaics differ widely in the extent and manner in which topography and bioclimate influence vegetation pattern (S1 Fig), management plans should be tailored to the elevation and geographic position of individual mosaics, with different management guidelines for the more strongly climatically determined mosaics (above 2000m). A better understanding of the role of fire in the maintenance of grasslands is essential [2,8].

The importance of local topography indicates that microclimate [7] regulates vegetation pattern in these mosaics. Projections of vegetation range shifts for these habitats should therefore account for variation in topography and its interaction with changing regional climate and disturbance regimes [7,21]. The results support the potential role of topographic heterogeneity in creating climatic microrefugia for vegetation within these mosaics that may allow species and plant community types to persist for longer periods of time. Hence, it is important to continue to protect forest-grassland mosaics in the Western Ghats and to increase coordinated research, monitoring and conservation efforts in these habitats.

## Supporting Information

S1 Fig Analysis of terminal nodes of classification tree. Stacked barplots showing the regional identity and vegetation community type of data points falling in each of the terminal nodes of the classification tree in Fig 3.
(PDF)
