# Supplementary material for: Topographic and Bioclimatic Determinants of the Occurrence of Forest and Grassland in Tropical Montane Forest-Grassland Mosaics of the Western Ghats, India
Source: PLoS One. 2015 Jun 29;10(6):e0130566. doi: 10.1371/journal.pone.0130566 (PMC4488301; doi:10.1371/journal.pone.0130566)
Supplement: S1 Fig — Stacked barplots showing the regional identity and vegetation community type of data points falling in each of the terminal nodes of the classification tree in Fig 3. (PDF) [file pone.0130566.s001.pdf]

Appendix S1: Stacked barplots showing the regional identity and community type of data points falling in each of the terminal nodes of the classification tree in Figure 3

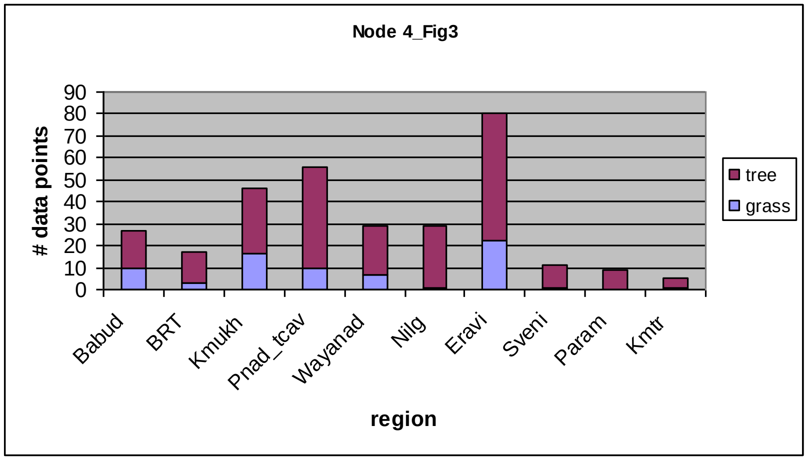

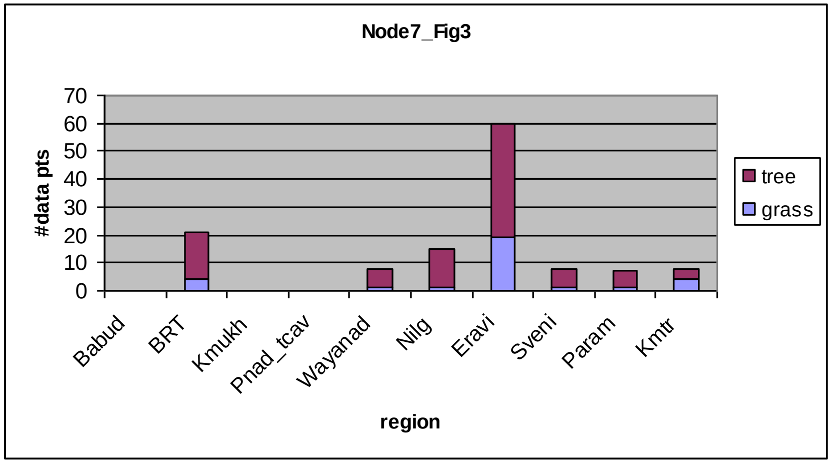

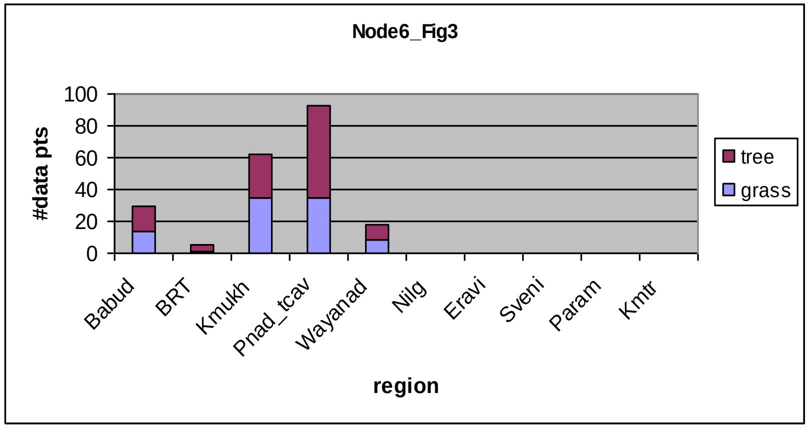

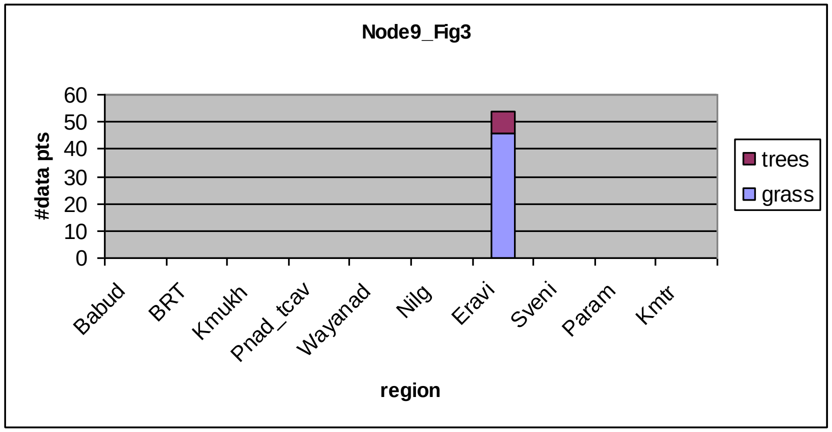

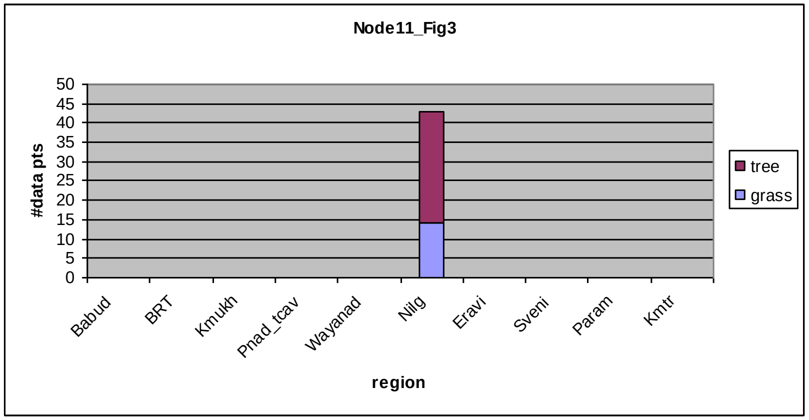

Node11_Fig3
50
45
40
35
30
25
20
15
10
5
0
#data pts
Babud
BRT
Kmukh
Pnad_tcav
Wayanad
Nilg
Eravi
Sveni
Param
Kmtr
region
tree
grass

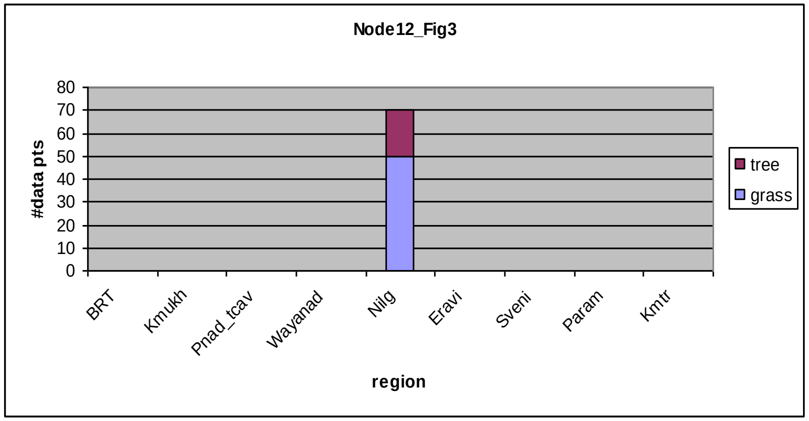

Node12_Fig3
80
70
60
50
40
30
20
10
0
#data pts
BRT
Kmukh
Pnad_tcav
Wayanad
Nilg
Eravi
Sveni
Param
Kmtr
region
tree
grass

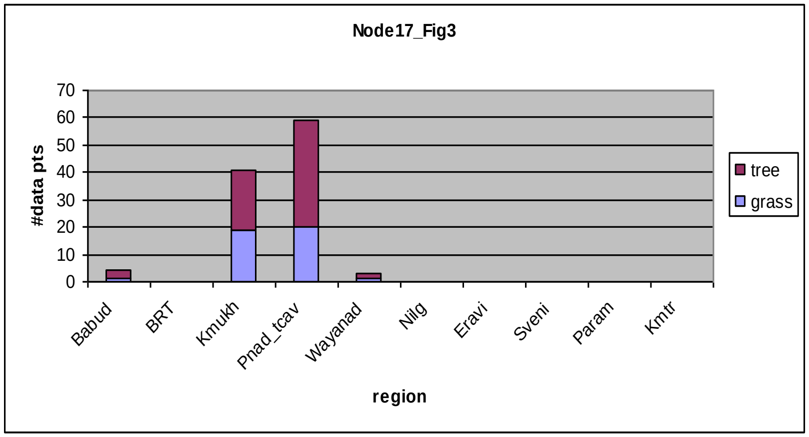

Node17_Fig3
70
60
50
40
30
20
10
0
#data pts
Babud
BRT
Kmukh
Pnad_tcav
Wayanad
Nilg
Eravi
Sveni
Param
Kmtr
region
tree
grass

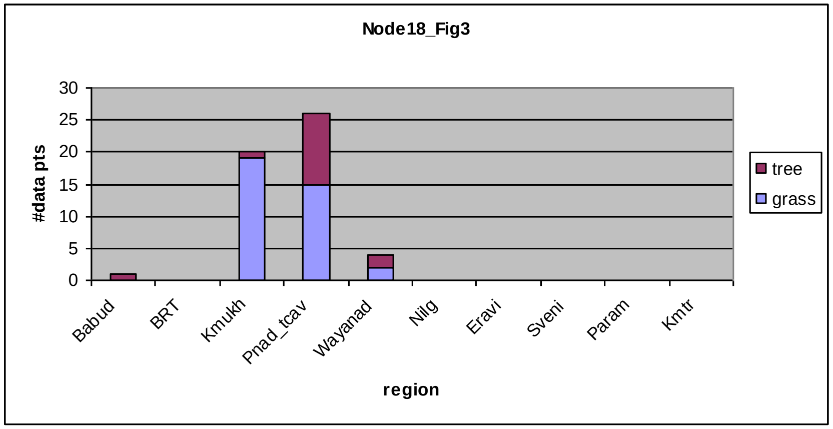

Node18_Fig3
30
25
20
15
10
5
0
#data pts
Babud
BRT
Kmukh
Pnad_tcav
Wayanad
Nilg
Eravi
Sveni
Param
Kmtr
region
tree
grass

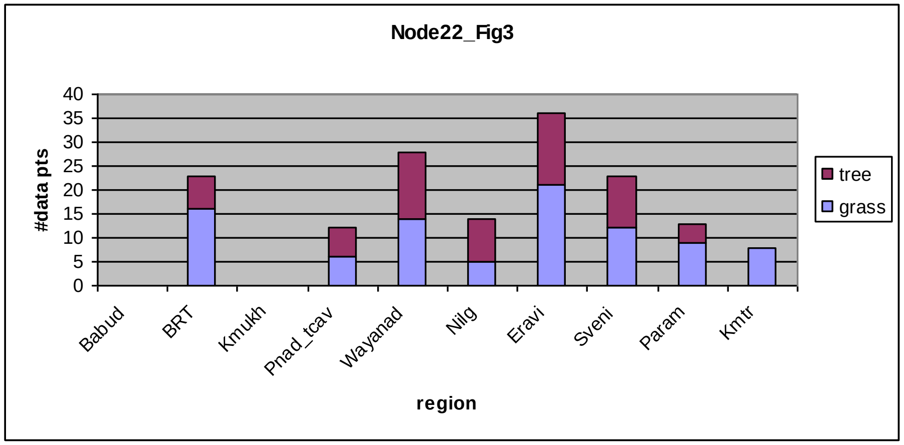
Node22_Fig3
#data pts
40
35
30
25
20
15
10
5
0
Babud
BRT
Kmukh
Pnad_tcav
Wayanad
Nilg
Eravi
Sveni
Param
Kmtr
region
tree
grass

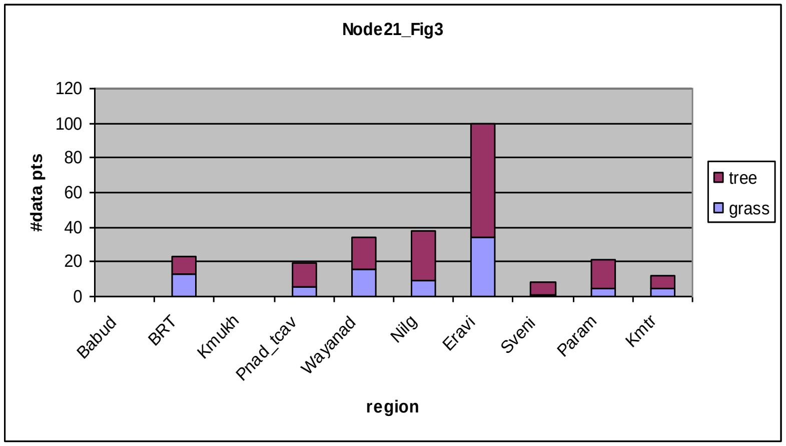
Node21_Fig3
#data pts
120
100
80
60
40
20
0
Babud
BRT
Kmukh
Pnad_tcav
Wayanad
Nilg
Eravi
Sveni
Param
Kmtr
region
tree
grass

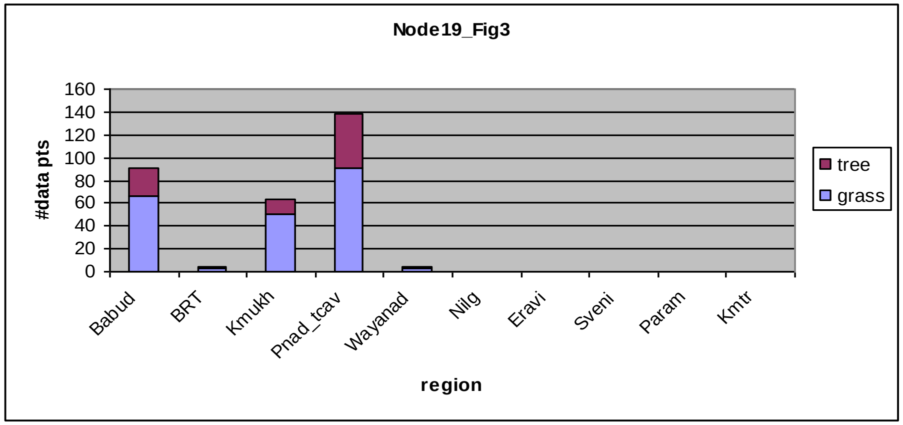
Node19_Fig3
#data pts
160
140
120
100
80
60
40
20
0
Babud
BRT
Kmukh
Pnad_tcav
Wayanad
Nilg
Eravi
Sveni
Param
Kmtr
region
tree
grass

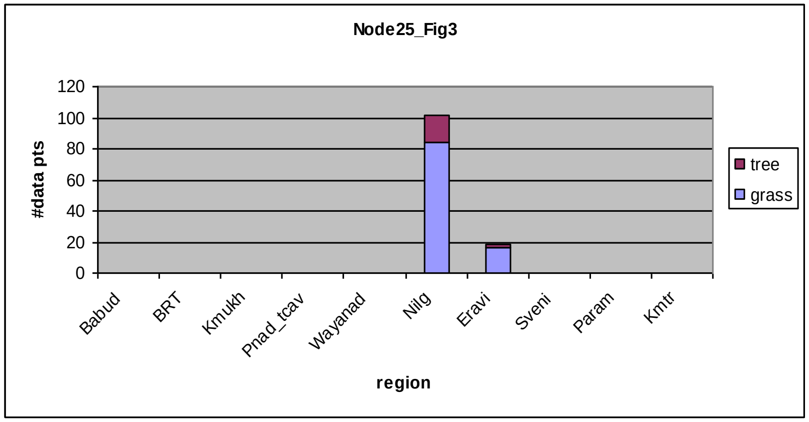
Node25_Fig3
#data pts
120
100
80
60
40
20
0
Babud
BRT
Kmukh
Pnad_tcav
Wayanad
Nilg
Eravi
Sveni
Param
Kmtr
region
tree
grass

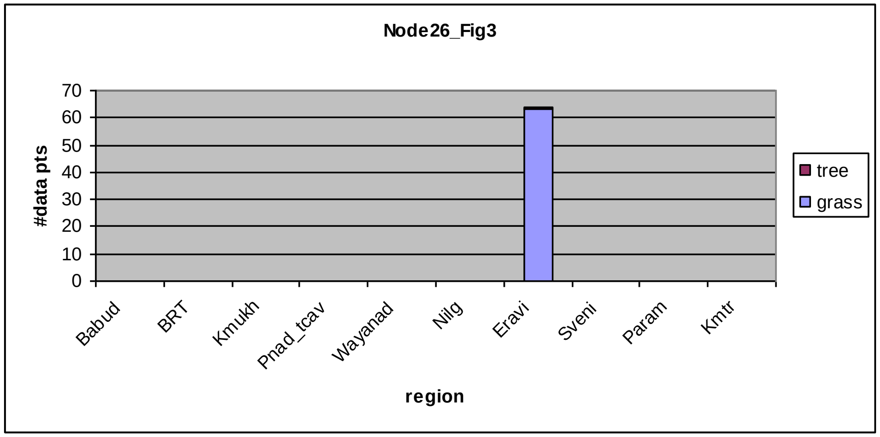

Node26_Fig3
70
60
50
40
30
20
10
0
#data pts
Babud
BRT
Kmukh
Pnad_tcav
Wayanad
Nilg
Eravi
Sveni
Param
Kmtr
region
tree
grass

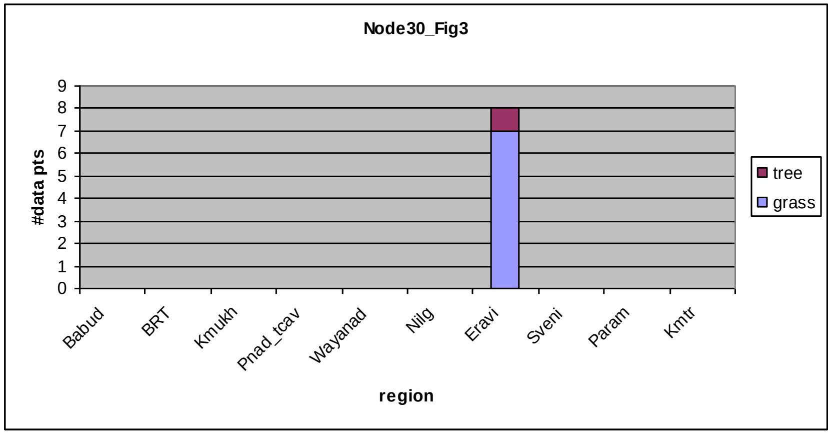

Node30_Fig3
9
8
7
6
5
4
3
2
1
0
#data pts
Babud
BRT
Kmukh
Pnad_tcav
Wayanad
Nilg
Eravi
Sveni
Param
Kmtr
region
tree
grass

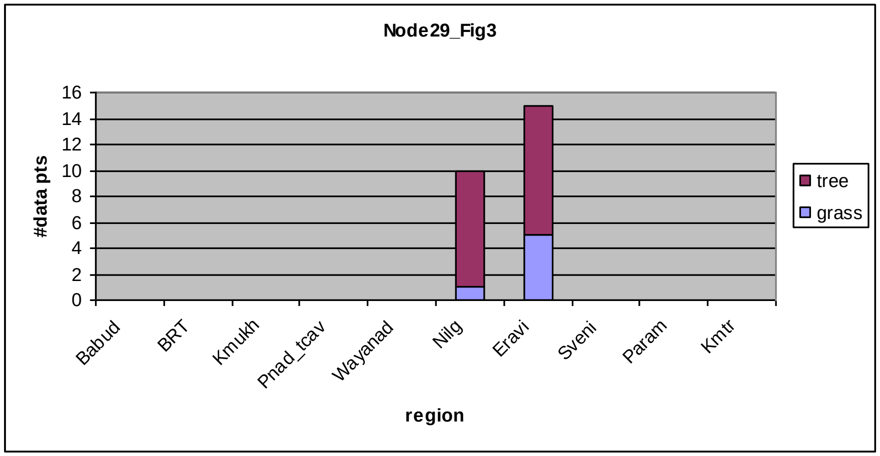

Node29_Fig3
16
14
12
10
8
6
4
2
0
#data pts
Babud
BRT
Kmukh
Pnad_tcav
Wayanad
Nilg
Eravi
Sveni
Param
Kmtr
region
tree
grass

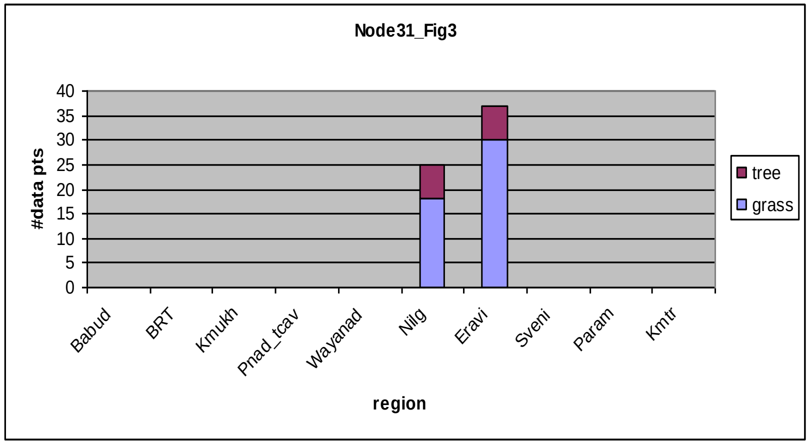

Node31_Fig3
40
35
30
25
20
15
10
5
0
#data pts
Babud
BRT
Kmukh
Pnad_tcav
Wayanad
Nilg
Eravi
Sveni
Param
Kmtr
region
tree
grass
